# The Effect of Dietary Oil Type and Energy Intake in Lactating Sows on the Fatty Acid Profile of Colostrum and Milk, and Piglet Growth to Weaning

**DOI:** 10.3390/ani9121092

**Published:** 2019-12-06

**Authors:** Anna Lavery, Peadar G. Lawlor, Helen M. Miller, Elizabeth Magowan

**Affiliations:** 1 Pig Development Department, Animal and Grassland Research Innovation Centre, Teagasc, Moorepark, P61 C996 Fermoy, Ireland; peadar.lawlor@teagasc.ie; 2 Faculty of Biological Sciences, University of Leeds, Leeds LS2 9JT, UK; h.m.miller@leeds.ac.uk; 3 Agri-Food and Bioscience Institute, Large Park, Hillsborough BT26 6DR, UK; elizabeth.magowan@afbini.gov.uk

**Keywords:** salmon oil, energy, phase feeding, lactation nutrition, piglet growth

## Abstract

**Simple Summary:**

Substituting soya oil with salmon oil in lactation diets improved sow milk yield and litter gain during the suckling period. Additionally, salmon oil tended to reduce pre-weaning mortality; therefore, its use in lactation diets should be further investigated. A phased dietary energy regimen increased sow lactation energy intake, but sow body condition and piglet growth performance to weaning were not improved.

**Abstract:**

This study investigated the effect of salmon oil in lactating sow diets and offering these diets in a phased dietary regimen to increase the energy density of the diet in late lactation. Sow and piglet productivity to weaning, the fatty acid profile of milk, piglet blood and tissues at weaning were the main parameters measured. Multiparous sows (*n* = 100) (Landrace × Large White) were offered dietary treatments from day 105 of gestation until weaning. Dietary treatments (2 × 2 factorial) included oil type (soya or salmon oil) and dietary regimen (Flat 14.5 MJ/kg DE diet offered until weaning or Phased 14.5 MJ/kg DE diet offered to day 14 of lactation then a second diet containing 15.5 MJ/kg DE offered from day 15 until weaning). Salmon oil inclusion increased the total proportion of n-3 fatty acids in colostrum (*p* < 0.001), milk (*p* < 0.001), piglet plasma (*p* < 0.01), adipose (*p* < 0.001), liver (*p* < 0.001) and muscle (*p* < 0.001). Increasing sow dietary energy level in late lactation increased the total n-3 fatty acids in milk (*p* < 0.001), piglet adipose (*p* < 0.01) and piglet muscle (*p* < 0.05). However, piglet growth to weaning did not improve.

## 1. Introduction

As the global demand for pork continues to rise, increasing the number of piglets weaned per litter at a good weaning weight represents a key challenge. Piglet weight at weaning is a major determinant of post-weaning performance [1]. However, sow milk yield is a limiting factor for the growth of nursing piglets [2]. The amount of milk needed for 1 g of live-weight gain increases as lactation progresses as the energy requirement for maintenance of piglets increases, with 317, 531 and 582 g/day of milk needed to maintain piglet live-weight in weeks 1, 2 and 3, respectively [3]. Therefore, considerable energy is needed to support milk production. A dietary digestible energy density of 14.05 MJ/kg DE is recommended for lactating sows of parity 2 and above with a litter size of 11.5 and a litter average daily gain of 190–270 g/day [4]. However, the average litter size in the European Union is currently 13.8 piglets born alive [5]; therefore, research into the energy requirements of modern prolific lactating sows is needed to better support the growth of piglets reared in these larger litters.

Increasing the energy density in the lactation diet should help increase energy intake during lactation to better match the demand of the suckling litter whilst minimising catabolism of maternal body reserves. Park et al. [6] found that a high energy diet (14.7 MJ/kg DE) in lactation reduced sow body weight and back-fat loss and increased piglet growth to weaning compared to a low energy diet (14.2 MJ/kg DE). Craig et al. [7] also reported an increase in litter average daily gain when a high energy diet (15.8 MJ/kg DE) was offered compared to a ‘normal’ diet (15.2 MJ/kg DE). However, feed intake in sows commonly plateaus in late lactation due to limited gut capacity. Therefore, increasing the dietary energy density during late lactation could help improve sow energy intake in late lactation. Achieving this could increase milk production and subsequently piglet growth in late lactation.

Oils are commonly included in pig diets due to their high energy availability compared to cereals and currently 6.0 g/day of the n-6 fatty acid linoleic acid (LA) is recommended for lactating sows [4]. Oil inclusion in late gestation and during lactation has been found to influence the fatty acid composition of milk, increase the output of fat and energy in milk and improve piglet gain from birth to weaning [8]. Whilst soya oil is commonly used in pig diets and represents a rich source of n-6 fatty acids, fish oils, such as salmon oil, contain a high level of n-3 fatty acids, which have been found to reduce pre-weaning mortality and improve the growth of suckling pigs [9,10]. Although the benefits of n-3 oil have been widely researched, there is currently no recommended inclusion level for n-3 fatty acids in sow diets. 

The objective of this study was to examine the effect of soya oil and salmon oil in sow lactation diets while increasing the energy density of the diet in late lactation on sow and piglet productivity to weaning as well as the fatty acid profile of milk, piglet blood and piglet tissues at weaning. Piglet growth, transfer of fatty acids from feed to milk and to piglets until weaning were investigated.

## 2. Materials and Methods

This study was conducted at the Agri-Food and Bioscience Institute, Hillsborough, Co. Down, Northern Ireland, from May 2017 to January 2018. This study was carried out under project license 2751 entitled “Manipulating the nutrition and environment of pigs to optimise and understand their productive performance and welfare” granted by the Department of Health, Social Services and Public Safety (DHSSPS) of Northern Ireland in accordance with the Animals (Scientific Procedures) Act 1986 (The Parliament of the United Kingdom, 1986).

Multiparous sows (parities two to nine; *n* = 100), with a mean parity 4.4 (SD = 3.21) were blocked according to parity, body condition score and body weight prior to being randomly allocated to treatment on day 105 of gestation. Sows were PIC F1 cross (Large White × Landrace) and Danish Duroc was the terminal sire used. For each batch of sows, artificial insemination was completed over a three day period. Each sow was inseminated twice over the three day period. There were 10 batches of animals with approximately 10 sows per batch. During the first 14 days of gestation, sows were kept in groups of four in free-access cubicles with a pen at the rear (space allowance 2.76 m^2^). After day 14, sows were moved to a large dynamic group of approximately 80 sows, where there were fed using an electronic sow feeder (Nedap Livestock Management, 7141 DC Groenlo, the Netherlands) until day 105 of gestation. Sows were offered 2.5 kg/day of the same barley-based diet (12.9 MJ/kg DE, 14.02 g/kg Crude Protein and 0.7 g/kg total Lysine) from weaning to day 85 of gestation and then 3.0 kg/day until they moved to the farrowing accommodation on day 105 of gestation.

Sows were moved to the farrowing accommodation on approximately day 105 of gestation and were housed in farrowing crates, with an enclosed heated creep area for the piglets at the front of each crate. The temperature of both the farrowing room and piglet creep areas was set electronically, with the ambient temperature set at 19 °C for farrowing and thereafter reduced gradually to 17.5 °C. The creep area was set at 30 °C and gradually reduced to 23 °C during the seven days post-farrowing. Sows were fed using wet and dry feeders and were offered 3 kg/day of their respective lactation diet until the day of farrowing, after which feed allowance was increased by 0.5 kg/day to appetite. Individual sow feed allowance was recorded, weighed and offered manually over two meals per day, with feed disappearance recorded as feed intake.

Sows were induced to farrow with 2 mL of Lutalyse (Zoetis Service LLC, Parsippany, NJ, USA) on day 114 of gestation. For each sow, total born, born alive, born dead and mummified pigs were recorded. Within the first 12 h of birth, piglets had their teeth clipped, tails docked and tail and umbilical area sprayed with iodine. Piglets were injected with 2 mL of an iron supplement (Uniferon, Virbac Ltd., Suffolk, UK) and given an ear tag to allow for individual identification throughout their lifetime. Cross-fostering was completed within 24 h after farrowing and occurred within oil treatments, with litters standardised to approximately 14 piglets in so far as possible. The weight and cause of all piglet mortalities were recorded. Piglets had free access to water from nipple drinkers and creep feed was not offered with sow troughs high enough to deter piglets from consuming sow feed. Piglets were weaned at approximately 28 days old. Sow weaning to service interval was recorded.

Dietary treatments commenced on day 105 of gestation, on entry to the farrowing crate. Dietary treatments were provided in a 2 × 2 factorial arrangement. The factors were: 1. Oil type (soya or salmon oil (Rossyew, Ltd., Greenock, UK) and 2. Dietary regimen Flat (14.5 MJ/kg DE, 17.0 g/kg CP, 1.2 g/kg Lys, diet offered for 28 days of lactation) or Phased (14.5 MJ/kg DE diet offered until day 14 of lactation and an immediate change to a second diet containing 15.5 MJ/kg DE, 17.0 g/kg CP, 1.3 g/kg Lys, offered from day 15 to day 28 of lactation). The feed was manufactured on site at the Agri-food and Bioscience Institute, Hillsborough (Northern Ireland) and diets were offered in meal form and the fatty acid composition of diets were analysed (Table 1).

Sows were weighed on day 105 of gestation and at weaning. Back-fat depth and body condition score were measured on day 105, 110 and 114 of gestation and on day one, seven, 14, 21 and 28 of lactation. Back-fat depth was measured at the P^2^ position (65 mm from the midline at the level of the last rib) with an ultrasonic back-fat scanner (Pig Scan-A-Mode back-fat scanner, SFK Technology, Herlev, Denmark). Body condition score was scored using a five-point scale and half scores were also used, with a score of one being when the sow was visually thin; hips and back bone very prominent and a score of five being when the sow was fat; it was impossible to feel hipbones and backbone. Sow empty body weight was calculated using the formula: sow empty weight (kg) = sow weight pre-farrowing, (day 105 (kg)) − (total number of piglets born × 2.28) [11]. Sow lactation efficiency was calculated by dividing sow energy input during lactation by total litter gain (kg) after cross-fostering, where energy input was calculated by adding the total energy intake from feed during lactation to the energy gained from sow weight lost during lactation (assuming every 1 kg loss = 12.5 MJ DE) [12].

Colostrum samples (*n* = 20/treatment) were obtained within four hours after farrowing had commenced and milk samples were collected on day 14 and 21 of lactation. To collect milk samples, piglets were prevented from suckling one hour prior to collection and 1 mL of oxytocin was administered intramuscularly in the sows’ neck. Approximately 80 mL of milk was obtained by hand from all mammary glands and stored at −20 °C pending analysis. Sow milk yield was calculated as piglet gain × 4.2 [13]. The first 48 h of each farrowing was recorded to allow litter suckling duration and frequency to be analysed. Videos were analysed for the first 24 h after farrowing was complete, which in this study was defined as after the expulsion of the placenta. The duration of each suckling bout and the frequency were recorded. A suckling bout was defined as more than 60% of the litter actively suckling and was finished when more than 60% of the piglets were no longer suckling or if the sow terminated suckling by rolling onto the udder or standing up. All piglets were individually weighed at birth, day one, five, seven, 14, 21 and 28 of lactation. Litter and piglet mean weight, average daily gain and coefficient of variation (CV) of litter weight were calculated. 

A sub sample of piglets (*n* = 10/treatment) were used for dissection. One pig of mean weaning weight per litter, balanced for sex, was selected for dissection on day 28 of lactation. Blood samples were obtained by jugular vein puncture into evacuated tubes. Serum and plasma samples were prepared from the blood samples by centrifugation for 15 min at 2500× *g* at 4 °C (Mistral 3000E centrifuge, MSE, Lower Sydenham, London, UK), with samples stored at −20 °C pending analysis. At weaning, selected pigs were euthanised by an injection of pentobarbital sodium (Dolethal, Vetoquinol UK Ltd., Buckinghamshire, UK). Once euthanised, pigs were individually scanned using a Dual Emission X-Ray Absorptiometry (DXA) scanner (Stratos DR Bone Densitometer, DMS, Mauguio, France) whole body scan to determine body and bone composition, i.e., total fat mass, total lean mass, bone mineral content and density. Each pig was then dissected, and the liver removed and weight recorded. Samples were macerated, vacuum packed and stored at −20 °C pending analysis. A 10 cm^2^ P2 muscle sample from the right side of the pig was also removed. The skin layer was removed and discarded. The subcutaneous fat layer was removed, and both the fat and muscle sample weights were recorded before samples were macerated, vacuum packed and stored at −20 °C pending analysis. 

Plasma, colostrum, milk, liver, muscle and fat samples stored at −20 °C were analysed for Fatty acid methyl esters (FAMEs). Plasma, colostrum and milk samples were prepared using a rapid total lipid extraction described by Bligh and Dyer [14]. Liver, muscle and fat samples were prepared as per the method of O’Fallon et al. [15]. Methylated samples were stored at −20 °C until they were analysed on the Gas Chromatograph (GC). The methylated extract (1 µL injection) were analysed on an Agilent 6890 GC with FID, a 7683 series injector and autosampler, with a CP-Sil 88,100 m 0.25 diameter column (Agilent, Cheadle, UK). Data acquisition was carried out using Openlab software (Agilent). Fatty acids reported include total saturated fatty acids, mono-unsaturated fatty acids (MUFA), poly-unsaturated fatty acids (PUFA), n-3 PUFAs; α-linolenic acid (ALA), eicosapentaenoic acid (EPA), docosapentaenoic acid (DPA), docosahexaenoic acid (DHA) and n-6 PUFAS; linoleic acid (LA) and arachidonic (ARA). Serum concentration of immunoglobulin G (IgG) was assayed using specific pig-ELISA kits (Bethyl Laboratories Inc., Universal Biologicals, Cambridge, UK). The IgG was quantified according to the manufacturer’s instructions. 

Sow, litter and piglet variables recorded until day 14 of lactation and colostrum and day 14 milk samples were analysed for the effect of oil treatment only. Sow, litter and piglet performance variables from day 15 until weaning as well as the fatty acid profile of day 21 milk samples, piglet blood plasma and tissues collected at weaning were analysed as per the 2 × 2 experimental arrangement, to determine the interactive effect between oil type and energy level as well as the direct effects of oil and energy. All continuous response variables and repeated measures were modelled using linear mixed model methodology. Binary and count variables were modelled using generalised linear mixed model methodology with a binomial distribution and logit link function for the binary variables and a Poisson distribution and logarithm link function for the count variables. In all analyses, parity and treatment were included as fixed effects, while day was included as an additional fixed effect where applicable. Where applicable, batch, day and nursing sow were used as additional random effects within the model. For each response, variable additional explanatory variables were fitted as fixed effects. A backwards elimination procedure was applied to these additional fixed effects for each response variable so that only variables that were significant at the (*p* < 0.05) level remained in the final model in each case. All models were fitted using residual maximum Likelihood in the statistical software package GenStat (18th edition, VSN Internal Ltd., Hemel Hampstead, UK). If differences detected were significant, comparisons between groups were conducted with the fisher’s least significant difference test. None of the data were deposited in an official repository. All data reside with the authors and data files can be requested.

## 3. Results

### 3.1. Diet Composition

While the actual analysis of diets revealed that DE and CP levels were higher than formulated, they were broadly in line with the target differences between treatments (Table 1). With regards the fatty acid composition of diets with different oil types, diets containing salmon oil had more saturated, MUFA and n-3 fatty acids and lower proportions of PUFA, n-6 fatty acids and n-6:n3 fatty acid ratio than diets containing soya oil (Table 1). However, the concentration of fatty acids in diets containing soya oil was similar irrespective of inclusion level of soya oil. Fatty acids below the detection limit (1.0 g/100 g total fatty acids) were represented in total saturated MUFA, PUFA, n-3 and n-6 calculations, and contributed approximately 2.0% to the fatty acid content in diets containing soya oil and between 4.0–5.0% of the fatty acid content in diets containing salmon oil.

### 3.2. Sow Characteristics

Since the phased energy regimen was not effective until day 15 of lactation, its impact is reported from day 15 only. There was no interaction between dietary oil type and energy regimen and no direct effects of oil type or energy regimen on sow back-fat depth or body condition score from day 21 to weaning (*p* < 0.05). Over the duration of the experiment, sow back-fat depth and body condition score were not affected by oil type (*p >* 0.05) but were influenced by day (*p* < 0.001). Sow back-fat depth decreased during lactation from 21.1 mm on day 1 of lactation to 17.9 mm at weaning. Sow body condition score decreased from day 105 of gestation until weaning, with body condition score on day 14 and 21 of lactation significantly lower than body condition score at day 105 and day 7 of lactation (*p* < 0.05).

There was no interactive effect between oil type and energy regimen on overall sow lactation feed intake (*p >* 0.05). Sow feed intake in the first and second week of lactation was not influenced by dietary oil type (*p >* 0.05), but sows offered a diet containing salmon oil ate 61.6kg in the third week of lactation (SEM = 1.23), 3.5 kg more feed compared to sows offered a diet containing soya oil (*p* < 0.05) (Table 2). Feed intake in the fourth week of lactation was unaffected by dietary oil treatment (*p >* 0.05). Dietary energy regimen did not affect sow feed intake during the third and fourth of lactation (*p >* 0.05). However, offering a diet with a higher energy level from day 15 of lactation to weaning increased sow energy intake in this period, from 1745 MJ DE to 1940 MJ DE (SEM = 43.5; *p* < 0.01) and by 228 MJ DE in overall lactation energy intake (SEM = 53.7; *p* < 0.01). Despite this, sow lactation efficiency was not improved (*p >* 0.05). Sow live weight at weaning and weaning to service interval were not affected by dietary treatment (*p >* 0.05).

There was no interactive effect between oil type and energy regimen on sow milk yield (*p >* 0.05). Sows offered diets containing salmon oil produced on average 15.3 L/day from day seven to 14 and 15.0 L/day from day 21 to 28 of lactation, which was 1.15 L/day (SEM = 0.39) and 1.73 L/day (SEM = 0.54) more milk, respectively, than sows offered lactation diets containing soya oil (*p* < 0.05). There was no effect of energy regimen on sow milk yield in late lactation (*p >* 0.05). Independent of dietary treatment, sow milk yield was affected by day of lactation (*p* < 0.001). Milk yield significantly decreased from 10.2 L/day on day one to 6.7 L/day on day five of lactation (SEM = 0.53; *p* < 0.05), but increased again to 12.0 L/day on day seven of lactation. Milk yield peaked at day 14, with an average milk yield of 14.6 L/day and plateaued to an average of 14.0 L/day between day 21 to 28 of lactation (*p >* 0.05).

### 3.3. Litter Performance

There was no interaction between oil type and energy regimen for any litter performance measures (*p >* 0.05). There was no effect of oil type on total born, born alive, number of piglets stillborn (*p >* 0.05). Mean total born and born alive were 15.7 (SEM = 0.32) and 14.1 (SEM = 0.33), respectively. As litters were standardised to 14 piglets within 24 h, the mean litter size after fostering was 13.8 (SEM = 0.16). There was a tendency (*p* = 0.06) for a reduced pre-weaning mortality rate for sows offered a diet containing salmon oil compared to sows offered a diet containing soya oil (9.87 versus 13.35%, respectively). The average number of piglets weaned per litter was 12.2 (SEM = 0.16) and pre-weaning mortality rate was 11.2% (SEM = 1.07). Mean total litter live-weight at birth was 19.6 kg (SEM = 0.47) and total litter weaning weight averaged 104 kg (SEM = 1.81) (Table 2). Litters from sows offered diets containing salmon oil during lactation had an increased litter weight gain of 0.5 kg/day from day seven to 14 (SEM = 0.12; *p* < 0.01) and 0.4 kg/day from day 21 to 28 of lactation (SEM = 0.02; *p* < 0.05), compared to litters from sows offered diets containing soya oil. The coefficient of variation (CV) of litter weight from birth to weaning was not influenced by sow dietary treatment (*p >* 0.05). Litter suckling duration and suckling frequency in the first 24 h post-farrowing did not differ between dietary oil types (*p >* 0.05). Dietary energy regimen did not influence litter growth to weaning (*p >* 0.05). As expected, day had a significant effect on litter growth to weaning (*p* < 0.001). Litter live-weight was not significantly different between birth and day one (*p >* 0.05) but thereafter, litter live-weight increased significantly as lactation progressed (*p* < 0.001). Similarly, litter average daily gain increased throughout lactation (*p* < 0.001), while within litter variation, as measured by the CV, in piglet weight decreased as lactation progressed from 22.4% at birth to 17.5% at weaning (*p* < 0.001).

### 3.4. Piglet Growth to Weaning

In the current study, a total of 1145 piglets were used to evaluate growth performance to weaning. There was no interaction between oil type and energy regimen on piglet live-weight or weight gain from day 14 to weaning (*p >* 0.05). Dietary oil type offered to sows did not influence piglet weight during the suckling period. Mean piglet birth weight was 1.35 kg (SEM = 0.010) and average piglet weight at weaning was 8.5 kg (SEM = 0.052). Piglet average daily gain from day five to seven (*p* < 0.001) was greatest for piglets from sows offered diets containing soya oil, with piglets gaining on average 80 g/day more compared to piglets from sows offered diets containing salmon. However, piglet average daily gain at any other time point and overall piglet gain from birth to weaning were unaffected by dietary oil type (*p >* 0.05). There was no effect of energy regimen on piglet growth performance to weaning (*p >* 0.05). When analysed as repeated measures, there was no interaction between oil type and energy regimen on piglet growth in late lactation (*p >* 0.05). Irrespective of maternal dietary treatment, piglet weight increased from birth to weaning (*p* < 0.001) and piglet average daily gain increased until day 14 and plateaued until weaning (*p* < 0.001).

### 3.5. Colostrum and Milk Fatty Acids

There was a significant interaction between oil type and energy regimen in late lactation on the fatty acid profile of milk collected on day 21 of lactation (*p* < 0.05) (Table 3). Offering sows a diet containing salmon oil and the phased energy regimen increased the proportions of the n-3 PUFAs C20:5, C22:5 and C22:6 (*p* < 0.001), and decreased proportions of the n-6 PUFA C18:2 (*p* < 0.001) compared to all other treatment groups. Therefore, offering sows the salmon oil and the phased energy regimen significantly increased the proportion of n-3 fatty acids (*p* < 0.001) and decreased the ratio of n6:n3 (*p* < 0.001), whereas the proportion of total PUFA (*p* < 0.001) and n-6 fatty acids was greatest in milk at day 21 from sows offered soya oil and the phased energy regimen. There was no interaction between oil type and energy regimen for the proportion of saturated and MUFA in sows’ milk at day 21 (*p >* 0.05). As expected, there was a significant interaction between dietary oil type and day of sampling on the fatty acid composition of colostrum and milk at day 14 and day 21. (Table 4). Although the total proportion of saturated and MUFA were unaffected by sow lactation dietary treatment and day (*p >* 0.05), the proportion of total PUFA (*p* < 0.05) and n-6 fatty acids (*p* < 0.001) were highest in colostrum (day 0) samples of sows offered diets containing soya oil. Offering sows a diet containing salmon oil significantly increased the proportion of n-3 fatty acids (*p* < 0.01) in colostrum (day 0), but the ratio of n6:n3 fatty acids (*p* < 0.001) was lowest in day 21 samples. 

### 3.6. Piglet Blood Plasma and Tissue Fatty Acids

There was a significant interactive effect between oil type and energy regimen on piglet blood plasma fatty acid profile at weaning (*p* < 0.05) (Table 5). Offering sows a soya oil diet and a phased energy regimen, increased the proportion of C18:2 (*p* < 0.05) and total n-6 fatty (*p* < 0.05) acids in piglet blood plasma at weaning compared to all other sow dietary treatments (*p* < 0.05).

#### 3.6.1. Adipose Tissue

There was an interactive effect between oil type and energy regimen on the proportion of fatty acids in piglet adipose tissue collected at weaning. Piglets at weaning from sows offered a diet containing soya oil and the phased energy regimen had a greater proportion of C18:2 (*p* < 0.01) than all other treatment groups (*p* < 0.05), but no difference was detected between piglets from sows offered diets containing salmon oil regardless of energy regimen (*p >* 0.05) (Table 5). Therefore, the total proportions of PUFA and n-6 fatty acids (both *p* < 0.01) were greatest in the adipose tissue of piglets from sows offered diets containing soya oil and phased dietary regimen. Feeding sows a diet containing salmon oil and the phased energy regimen increased the proportion of C20:5 (*p* < 0.01), C22:5 (*p* < 0.05), C22:6 (*p* < 0.05) in the adipose tissue of piglets compared to all other treatment groups (*p* < 0.05). However, the total proportions of MUFA and n-3 fatty acids in piglet adipose tissues were not influenced by sow dietary treatment (*p >* 0.05). 

#### 3.6.2. Liver Tissue

There was a significant interactive effect between oil type and energy regimen on the fatty acid composition of piglet liver tissue at weaning (Table 5). Piglets at weaning from sows offered a diet containing soya oil and the phased energy regimen had an increased proportion of C18:2 (*p* < 0.001) in liver tissue compared to piglets from sows offered diets containing salmon oil. The total proportions of PUFA and n-6 fatty acids (both *p* < 0.01) were greatest in the liver tissue of piglets from sows offered diets containing soya oil and phased dietary regimen compared to all other treatment groups (*p* < 0.05). Piglets from sows offered diets containing salmon oil and the phased energy regimen had greater proportions of C22:5 (*p* < 0.01) and total MUFA (*p* < 0.01) in liver tissue compared to all other treatment groups, with the exception of total MUFA, which was not different for piglets from sows offered salmon oil and flat energy regimen in lactation (*p >* 0.05).

#### 3.6.3. Muscle Tissue

There was a significant interactive effect between sow dietary oil type and energy regimen on the fatty acid profile of piglet muscle tissue collected at weaning (Table 5). Similar to adipose and liver tissue, muscle of piglets from sows offered a diet containing soya oil and a phased energy regimen had a greater proportion of C18:2 than all other treatment groups (*p* < 0.05). The total proportion of the fatty acid C22:6 and total n-6 (*p* < 0.05) were greatest in the muscle of piglets from sow offered diets containing salmon oil and the phased energy regimen, but the total proportions of saturated, MUFA, PUFA, n-3 and n6:n3 ratio in piglet muscle were unaffected (*p >* 0.05).

### 3.7. Piglet Serum IgG Concentration and Body Composition at Weaning

There was no effect of maternal lactation dietary treatment on IgG concentrations in piglet serum collected at weaning (*p >* 0.05). The average IgG concentration of piglet serum was 66.4 mg/mL (SEM = 10.5), with IgG concentrations ranging from 11.9 to 289.3 mg/mL. There was no interactive effect of oil type or energy regimen offered to sows on piglet bone or body composition measures (*p >* 0.05). Piglets born to sows offered the phased energy regimen had greater total bone area than piglet born to sows offered the flat energy regimen (420.3 versus 402.9 cm^2^, respectively). There was no direct effect of dietary oil type or energy regimen offered to sows during lactation on any other piglet bone or body composition measures recorded, i.e., bone mineral density, body fat, lean mass (*p >* 0.05).

## 4. Discussion

The benefits of increasing sow lactation dietary energy density and salmon oil inclusion in lactation diets have previously been reported separately, but this is the first time that both have been investigated simultaneously. There was no interactive effect between oil type and energy regimen on sow or piglet performance measures. This result is surprising, as it was expected that increasing the proportion of n-3 fatty acids in the lactation diet and increasing dietary energy density in late lactation would increase piglet growth through improved milk yield [10,16]. However, there were interactive effects observed which affected the proportion of fatty acids in piglet tissues and blood plasma collected at weaning as well as in sow milk samples. As expected, the proportion of n-3 fatty acids increased with increasing salmon oil inclusion in the phased energy diet, but the proportion of n-6 fatty acids did not increase with increasing soya oil inclusion in the phased diet. This is likely due to the contribution of n-6 fatty acids from the cereal ingredients. It is proposed that the key driver for the interactive effects seen in this study with regard to n-6 fatty acids was due to a change in energy balance causing sows offered the soya oil diet to mobilise body reserves and change the proportion of fatty acids seen in samples as the proportion of fatty acids in soya oil diets were similar irrespective of oil inclusion level.

Overall feed intake or energy intake did not differ between oil treatments in this study. Salmon oil could have reduced feed intake as oils high in unsaturated fatty acids can be prone to lipid peroxidation, but regular analyses of the peroxidation status of both the salmon oil and soya oil confirmed that peroxidation was minimal in the trial diets (<10 mEq/kg). The present study found that salmon oil in sow lactation diets tended to decrease pre-weaning mortality rate compared to soya oil (9.9% versus 13.4%, respectively). Similarly, Rooke et al. [9] reported that although salmon oil fed to sows during gestation reduced piglet birth weight, pre-weaning mortality was reduced from 11.7 to 10.2% by reducing the number of piglet deaths due to crushing. On the contrary, Smit et al. [17] found that supplementing sows with an n-3 PUFA marine oil did not reduce the pre-weaning mortality rate of low birth weight litters. In the current study, dietary oil type did not affect piglet birth weight or litter suckling duration or frequency, piglet body composition or blood serum IgG concentration at weaning. This suggests that improvements in piglet pre-weaning mortality were not mediated through increases in piglet vigour at birth, fat deposition or immunity at weaning. However, as piglet colostrum intake and cause of death were not explored in the current study, further investigation is required to fully understand how dietary salmon oil inclusion reduced piglet mortality.

Previous research has suggested supplementing sows with fish oil during lactation may accelerate the maturation of the piglet immune system [18]. However, in agreement with Leonard et al. [19], the present study observed no effect of oil type on piglet IgG blood serum concentrations at weaning. On the contrary, Rooke et al. [20] reported piglet IgG concentrations at weaning tended to be higher for progeny from sows supplemented with tuna oil. However, the authors concluded that piglet IgG concentration on day 28 of lactation is positively related to IgG levels at day seven, which are positively correlated to passively acquired IgG levels absorbed from sow colostrum. Indeed, as piglet IgG concentration in the present study varied greatly between individuals, it is likely a reflection of maternal IgG levels and resulting colostrum intake. In this study, salmon oil inclusion in the sow lactation diet increased litter average daily gain in the second and fourth week of lactation, but overall, there was no effect on litter weaning weight. This is consistent with the results of Fritsche et al. [21] and Taugbøl et al. [22], who reported no effect of n-3 PUFA supplementation to sows from day 107 of gestation to weaning on piglet or litter weaning weight. However, some studies have reported increased piglet growth until the end of the nursery period when sows are supplemented with n-3 oils in gestation and lactation [10,23,24]. Therefore, a longer period of n-3 supplementation to sows may have been needed before a positive effect on piglet growth is observed. Unfortunately, this study terminated at weaning, thus, any positive effect that may have occurred post-weaning was not recorded.

With current increases in litter size, it is important to maximise sow milk yield, as it is a limiting factor in the growth of suckling piglets [2]. However, nutritional strategies to improve sow milk yield have varying degrees of success. Lauridsen and Danielsen [8] reported no effect of oil inclusion or dietary oil type included on sow milk production. Contrary to this, the present study found that diets containing salmon oil increased sow milk yield on day 14 by 7.5% and day 28 by 11.6% with overall milk yield being 10% greater compared to sows offered diets containing soya oil, without negative effects on sow body reserves. Indeed, Lee et al. [25] found that supplementing sows with conjugated linoleic acid (CLA) increased sow milk yield by 10% compared to soya oil fed sows. Although the exact mechanism of how oil type effects milk production is unknown, previous research supplementing lactating dairy cows with CLA suggest there may be shift in energy partitioning towards milk synthesis [26], thereby conserving body reserves.

As expected, the fatty acid profile of sow colostrum and milk can be attributed to sow dietary oil treatment. In agreement with the findings of Lauridsen and Danielsen [8], this study found that DHA, DPA and EPA were greater in milk from sows fed salmon oil, reducing the overall ratio of n6:n3 fatty acids. Similarly, Arbuckle and Innis [27] reported fish oil inclusion increased milk DHA and EPA, but no difference in ARA was detected, while in the present study, salmon oil decreased ARA in sows’ milk. In the current study, EPA levels increased as lactation progressed and initially DPA and DHA decreased from colostrum to day 14 but increased again on day 21. In this study, sows were offered a phased energy regimen, which increased the energy density of the diet through an increase in the oil content; this likely explains the increased proportion of fatty acids in day 21 milk compared to day 14 milk. As maternal dietary fats alter the fatty acid profile of milk, this subsequently influences the proportion of fatty acids in the plasma of suckling piglets. Fritsche et al. [21] reported EPA and total n-3 fatty acids in piglet plasma increased with increasing age when sows were supplemented with fish oil from day 107 until weaning, suggesting piglets efficiently absorbed n-3 fatty acids from the sows’ milk. Although in the current study, no difference was detected between the levels of EPA in piglet plasma, DHA levels and the proportion of n-3 fatty acids increased, reducing the ratio of n6:n3 fatty acids in piglet blood plasma at weaning when sows were fed salmon oil diets. Also in the present study, salmon oil inclusion increased the level of DHA in piglet adipose, liver and muscle tissue at weaning, but C18:3 was greater in the tissues of piglet from sows offered lactation diets containing soya oil. The accumulation of C18:3 (ALA) in the tissues of piglets from sows fed soya oil diet may be attributed to a less efficient conversion of ALA to EPA and DHA, which is estimated to only be 10–15% efficient in the human liver [28].

There is little information in the literature regarding how n-3 fatty acids in sow diets influence body composition in offspring. In a review by Muhlhausler et al. [29], n-3 long chain PUFA supplementation of rodents in gestation and lactation resulted in a lower body fat mass in progeny, while there are conflicting results regarding fat deposition in pigs in response to n-6 supplementation [30,31,32]. In the present study, total piglet body fat and lean mass as well as estimated visceral and subcutaneous tissue area at weaning were unaffected by maternal dietary oil treatment during lactation. This might not be unexpected since both n-6 and n-3 fatty acids can influence body composition in the pig. It is thought long chain PUFAs may affect prostaglandin E2 production which is important in bone metabolism. Piglet bone mineral content and density were also unaffected by sow dietary treatment in the current study. This response was not unexpected as dietary ARA: DHA ratio level was on average 0.06:0.94 g/100 g total fat, while Mollard et al. [33] reported elevated bone mass of piglets with a dietary ARA: DHA ratio of 0.5:0.1 g/100 g total fat. Overall the substitution of salmon oil, for soya oil, in sow lactation diets increased the proportion of n-3 fatty acids in colostrum, milk, piglet blood plasma and tissues collected at weaning. Nonetheless, improvement in many of the parameters of interest in sows and/or piglets was not observed. However, dietary salmon oil tended to reduce piglet mortality and increase sow milk yield and for these reasons its use in sow lactation diets certainly warrants further investigation.

Oils are often used to increase the energy density of lactation diets and dietary fat can increase the palatability of diets [34]. Although sow lactation feed intake was not affected by increasing dietary energy density in this study, as expected, dietary energy intake in late lactation and overall lactation increased when dietary energy was increased in the final 14 days of lactation. The energy requirements for the lactation diets used in this study were calculated using the requirement tables in the British Society of Animal Science (BSAS) Nutrient Requirements Standards for Pigs [35]. From this, the energy requirements for a sow to rear 14 pigs to 8.5 kg at weaning would be 112.8 MJ/day DE. Although the number of piglets weaned in the present study were lower than expected (average. 12.2), sows offered the phased energy regimen achieved an average intake of 116.1 MJ DE per day, which enabled sows to rear piglets to the target weight (8.6 kg). However, overall total litter weight weaned was not influenced by treatment, which may be a result of high sow feed intake, as regardless of dietary treatment, average sow feed intake in the present study (7.1 kg/day) was much higher than that found in commercial herds [36].

Sow back-fat depth and body condition score in this study decreased as lactation progressed but were not affected by dietary treatment. It had been hypothesised that sow weight and body condition loss would be reduced as energy intake increased during lactation as a result of feeding the energy dense diet [37]; however, this was not the case. This is supported by Eissen et al. [38], who reported that additional feed intake in lactation was not used efficiently by sows nursing larger litters (≥11 pigs), as neither sow body condition loss was reduced or piglet weight gain was increased. Nonetheless, lactation weight loss was <10% in our study which should not have been sufficient to reduce subsequent reproductive performance [39]. As mentioned, sow milk yield is a limiting factor in the growth of nursing pigs and piglet milk intake increases with increasing body weight as maintenance energy requirements increase [3]. Therefore, the effect of maternal diet on milk production increases as lactation progresses [40]. Previous research on increasing sow milk yield by increasing dietary energy density was not successful [41]. Similarly, in the present study, increasing sow energy intake in late lactation did not increase sow milk yield as measured indirectly from piglet growth, with sow milk yield appearing to plateau on day 14, which is in agreement with previous research [41]. In the present study, the fatty acid composition of milk at day 21 of lactation, as well as of piglet plasma and tissues at weaning were affected by dietary energy density. Reducing dietary energy density during lactation can cause some fatty acids to be selectively stored and then mobilsed from fat deposits. Indeed, Beyer et al. [42] reported higher concentrations of C16:0, a saturated fat found at high concentrations in sow fat, in the milk fat of sows when they were fed at 80% of their ME requirement during lactation. In the current study, total saturated fat levels were higher in milk sampled at day 21, from sows offered the flat energy regimen during lactation. As sow back-fat depth did not differ in response to the dietary energy density provided, it is most likely that the fatty acids were directly incorporated from blood lipids, which are directly influenced by diet [43]. In the present study total bone area was found to be greater for piglets from sows offered the phased dietary regimen, which may be a result of reduced n6:n3 ratio in the sows’ milk, as previous research has suggested that increasing dietary n-3 fatty acids and reducing the ratio of n6:n3 fatty acids can improve the bone formation in rats and piglets [44,45]. Offering sows a phased dietary energy regimen to increase energy intake in lactation influenced the fatty acid profile of milk, piglet plasma and tissues, but sow body condition and piglet growth to weaning was not improved by this practice. This suggests that feeding a higher energy density diet in late lactation does not improve piglet performance when average sow feed intake is already high as was the case in the current study (7.1 kg/day or 106.5 MJ/kg/day DE with dietary energy level of 15.0 MJ/kg DE).

## 5. Conclusions

Overall, there was no synergistic effect between oil type and dietary energy regimen on sow or piglet productivity. The inclusion of salmon oil in sow lactation diets did not improve sow body condition, or piglet growth performance to weaning when compared to soya oil. However, there was a tendency for salmon oil to decrease piglet mortality and increase sow milk yield, and therefore, salmon oil in lactation diets for sows should be further investigated. Offering sows a phased feeding regimen did not benefit piglet growth to weaning compared with a flat feeding regimen. Sow lactation feed intake was already very high in this experiment, and this may help explain the lack of treatment effect found here. The findings from this study suggest that when sow lactation feed intake is high, a single diet with a digestible energy density of 15.0 MJ/kg DE will support modern multiparous sows and at a litter gain of 84.4 kg during lactation (i.e., 12 piglets weaned at. 8.5 kg at 28 days old).

## Figures and Tables

**Table 1:** Ingredients, formulated, actual and fatty acid analysis of experimental diets on a fresh basis (%).

Item	Soya Oil		Salmon Oil	
	Flat ^1^	Phased ^2^	Flat ^1^	Phased ^2^
Ingredient, %				
Wheat	28.2	30.0	28.2	30.0
Maize	39.99	33.8	39.99	33.8
Soya	16.7	16.6	16.7	16.6
Full fat soya	10.0	10.0	10.0	10.0
Soya oil	1.79	5.98	-	-
Salmon oil	-	-	1.79	5.98
Lysine	0.12	0.27	0.12	0.27
Mineral and Vitamin premix ^3^	3.20	3.20	3.20	3.20
Formulated				
Dry matter (%)	84.8	85.4	84.8	85.4
Crude Protein (%)	17.0	17.0	17.0	17.0
Crude Fibre (%)	2.88	2.80	2.88	2.80
Oil A (%)	5.67	9.62	5.67	9.62
Digestible Energy (MJ/kg)	14.5	15.5	14.5	15.5
Total Lysine (%)	1.20	1.30	1.20	1.30
Ash (%)	5.00	5.00	5.00	5.00
Calcium (%)	0.67	0.67	0.67	0.67
Phosphorus (%)	0.55	0.54	0.55	0.54
Actual				
Dry matter (%)	87.3	88.1	87.4	88.3
CP (%)	17.7	18.0	18.0	17.4
CF (%)	2.20	1.95	2.10	1.90
Oil A (%)	5.54	9.56	5.52	10.22
DE (MJ/kg)	15.0	15.9	15.0	15.9
Total Lysine (%)	1.19	1.33	1.23	1.29
Ash (%)	4.75	4.80	4.85	5.00
Calcium (%)	0.68	0.73	0.68	0.79
Phosphorus (%)	0.49	0.46	0.46	0.45
Fatty Acids ^4^				
Total saturated ^5^	16.4	16.0	17.3	17.9
Total MUFA ^6^	24.1	24.8	30.7	38.5
Total PUFA ^7^	53.8	53.2	46.4	37.8
Total n-3 PUFA ^8^	5.71	6.11	7.00	8.78
C18:3 (n-3)	5.60	6.03	5.43	5.45
C22:6 (n-3)	<1.00	<1.00	1.11	2.38
Total n-6 PUFA ^9^	53.7	53.1	44.9	34.6
C18:2 (n-6)	53.7	53.0	44.4	33.5
n-6:n-3 ^10^	9.41	8.69	6.42	3.94

^1^ 14.5 MJ/kg DE diet offered for 28 d of lactation. ^2^ 14.5 MJ/kg DE diet offered until d14 of lactation, 15.5 MJ/kg DE diet offered from d15 to 28 of lactation. ^3^ Premix provided (per tonne of finished feed) 8 MIU vitamin A, 2 MIU Vitamin D_3_ 750 gm Methionine, 1250 gm Threonine, 2800 gm Lysine, 1 gm Iodine from Calcium Iodate, 0.2 gm Selenium from Sodium selenite, 80 gm Iron from Ferrous Sulphate, 30 gm Manganese from Manganous Oxide, 12 gm Copper from Cupric Sulphate, 80 gm Zinc from Zinc Oxide, 125 gm Antioxidant from BHA/BHT. Sourced from Devenish Nutrition Ltd., Belfast, UK. ^4^ Fatty acids are reported as g/100 g of total fatty acids with a reporting limit of 1 g/100g. Values presented are mean percentages of total lipid fraction from two determinations extracted from bulked samples of diet. ^5^ Total saturated-saturated fatty acids. ^6^ Total MUFA-monounsaturated fatty acids. ^7^ Total PUFA-polyunsaturated fatty acids. ^8^ Total n-3 PUFA-omega 3 fatty acids polyunsaturated fatty acids. ^9^ Total n-6 PUFA-omega 6 fatty acids polyunsaturated fatty acids. ^10^ n-6: n-3 ratio of omega 6 fatty acids to omega 3 fatty acids.

**Table 2:** The effect of oil type in sow lactation diets and energy level in late lactation on sow lactation feed (kg) and energy intake (MJ DE) and litter growth performance from birth to weaning.

Variable	Oil Type				Energy Regimen			
	Soya	Salmon	SEM ^1^	*p*	Flat ^2^	Phased ^3^	SEM	*p*
Feed Intake, kg								
Week 1	30.23	29.53	0.700	0.482	-	-	-	-
Week 2	49.76	50.07	0.916	0.813	-	-	-	-
Week 3	58.07	61.55	1.226	0.048	59.29	60.33	1.220	0.552
Week 4	61.94	64.31	2.133	0.435	61.23	65.01	2.131	0.226
Day 1–14	80.01	79.58	1.376	0.829	-	-	-	-
Day 15–28	119.9	125.6	2.919	0.172	120.3	125.1	2.911	0.256
Total	200.0	205.3	3.634	0.315	199.1	206.2	3.622	0.170
Energy Intake, MJ DE								
Day 1–14	1160	1154	19.96	0.829	-	-	-	-
Day 15–28	1798	1886	43.59	0.161	1745	1940	43.48	0.002
Day 1–28	2961	3042	53.89	0.300	2887	3115	53.71	0.004
Litter Weight, kg								
Birth	20.89	21.65	0.425	0.209	-	-	-	-
Live-born	19.18	20.03	0.542	0.269	-	-	-	-
Day 1	19.53	20.44	0.484	0.190	-	-	-	-
Day 14	54.72	57.51	1.442	0.174	-	-	-	-
Day 21	78.76	82.06	1.989	0.244	79.16	81.36	1.989	0.518
Day 28	102.5	105.5	1.884	0.275	102.4	105.5	1.867	0.254
Litter ADG, kg/day								
Week 1	1.74	1.49	0.162	0.278	-	-	-	-
Week 2	3.33	3.81	0.116	0.004	-	-	-	-
Week 3	3.41	3.53	0.120	0.493	3.40	3.55	0.120	0.380
Week 4	3.15	3.56	0.128	0.024	3.28	3.44	0.127	0.372
Day 1–28	2.94	3.03	0.061	0.301	2.94	3.02	0.060	0.387

^1^ SEM—standard error of the mean. ^2^ 14.5 MJ/kg DE diet offered for 28 d of lactation. ^3^ 14.5 MJ/kg DE diet offered from day 1 to 14 of lactation and 15.5 MJ/kg DE diet offered from day 15 to 28 of lactation.

**Table 3:** Fatty acid composition (g/100g total fatty acids) of sow milk samples collected on day 21 of lactation after offering sows diets containing soya or salmon oil and a low or phased dietary regimen during lactation.

Fatty Acid ^1^	Soya Oil		Salmon Oil		SEM ^4^	Oil	Energy	Oil × Energy
	Flat ^2^	Phased ^3^	Flat	Phased		*p*	*p*	*p*
Total saturated ^5^	40.4	38.2	42.6	39.8	0.926	0.045	0.009	0.713
Total MUFA ^6^	37.7	34.4	37.8	37.3	0.873	0.088	0.038	0.099
Total PUFA ^7^	21.6 ^b^	27.3 ^c^	19.3 ^a^	22.4 ^b^	0.609	<0.001	<0.001	0.038
Total n-3 PUFA ^8^	2.26 ^a^	2.93 ^b^	2.89 ^b^	5.07 ^c^	0.128	<0.001	<0.001	<0.001
C18:3 (n-3)	1.83	2.45	1.75	2.43	0.075	0.490	<0.001	0.713
C20:5 (n-3)	0.05 ^a^	0.06 ^a^	0.25 ^b^	0.70 ^c^	0.021	<0.001	<0.001	<0.001
C22:5 (n-3)	0.18 ^a^	0.2 ^a^	0.34 ^b^	0.66 ^c^	0.024	<0.001	<0.001	<0.001
C22:6 (n-3)	0.07 ^a^	0.09 ^a^	0.42 ^b^	1.10 ^c^	0.04	<0.001	<0.001	<0.001
Total n-6 PUFA ^9^	19.4 ^b^	24.4 ^c^	16.4 ^a^	17.3 ^a^	0.54	<0.001	<0.001	<0.001
C18:2 (n-6)	18.4 ^b^	23.4 ^c^	15.5 ^a^	16.3 ^a^	0.534	<0.001	<0.001	<0.001
C20:4 (n-6)	0.36	0.35	0.32	0.28	0.012	<0.001	0.066	0.391
n6:n3 ^10^	8.65 ^c^	8.30 ^c^	5.72 ^b^	3.45 ^a^	0.158	<0.001	<0.001	<0.001

^1^ Milk fatty acids are reported as g/100g of total fatty acids with a reporting limit of 0.01 g/100 g. ^2^ 14.5 MJ/kg DE diet offered for 28 d of lactation. ^3^ 14.5 MJ/kg DE diet offered from day 1 to 14 of lactation and 15.5 MJ/kg DE diet offered from day 15 to 28 of lactation. ^4^ SEM—standard error of the mean. ^5^ Total saturated-saturated fatty acids. ^6^ Total MUFA-monounsaturated fatty acids. ^7^ Total PUFA-polyunsaturated fatty acids. ^8^ Total n-3 PUFA-omega 3 polyunsaturated fatty acids. ^9^ Total n-6 PUFA-omega 6 polyunsaturated fatty acids. ^10^ n-6: n-3-ratio of omega 6 fatty acids to omega 3 fatty acids. ^a,b,c^ Means with different superscripts within a row are different (*p* < 0.05).

**Table 4:** The effect of sow dietary oil type and day of sampling on the fatty acid composition (g/100 g total fatty acids) of sow colostrum (d0), day 14 and 21 (d14, d21) milk samples.

Fatty Acid ^1^	Soya Oil		Salmon Oil				SEM ^3^	Oil	Day	Oil × Day
	D0 ^2^	D14 ^2^	D21 ^2^	D0	D14	D21		*p*	*p*	*p*
Total saturated ^4^	26.5	41.5	39.3	27.5	40.8	41.2	0.556	0.013	<0.001	0.111
Total MUFA ^5^	31.1	36.9	36	33.1	39.1	37.7	0.625	0.002	<0.001	0.859
Total PUFA ^6^	42.3 ^e^	21.5 ^b^	24.5 ^c^	39.2 ^d^	19.9 ^a^	20.8 ^a,b^	0.582	<0.001	<0.001	0.04
Total n-3 PUFA ^7^	4.32 ^c^	2.29 ^a^	2.60 ^a,b^	5.03 ^d^	2.90 ^b^	3.98 ^c^	0.118	<0.001	<0.001	0.006
C18:3 (n-3)	3.22	1.85	2.15	3.13	1.79	2.09	0.065	0.181	<0.001	0.959
C20:5 (n-3)	0.08 ^a,b,c^	0.06 ^a,b^	0.06 ^a^	0.09 ^a,c^	0.22 ^d^	0.47 ^e^	0.019	<0.001	<0.001	<0.001
C22:5 (n-3)	0.57 ^c^	0.19 ^a^	0.20 ^a^	0.84 ^d^	0.35 ^b^	0.50 ^c^	0.021	<0.001	<0.001	<0.001
C22:6 (n-3)	0.23 ^b^	0.08 ^a^	0.09 ^a^	0.71 ^d^	0.40 ^c^	0.76 ^d^	0.035	<0.001	<0.001	<0.001
Total n-6 PUFA ^8^	38.0 ^e^	19.2 ^b^	21.9 ^c^	34.1 ^d^	17.0 ^a^	16.8 ^a^	0.511	<0.001	<0.001	<0.001
C18:2 (n-6)	35.5 ^e^	18.3 ^b^	20.9 ^c^	31.7 ^d^	16.0 ^a^	15.9 ^a^	0.503	<0.001	<0.001	<0.001
C20:4 (n-6)	1.09	0.38	0.35	1.01	0.36	0.3	0.02	<0.001	<0.001	0.065
n6:n3 ^9^	8.79 ^e^	8.4 ^d^	8.45 ^d,e^	6.80 ^c^	5.94 ^b^	4.57 ^a^	0.141	<0.001	<0.001	<0.001

^1^ Colostrum and milk fatty acids are reported as g/100 g of total fatty acids with a reporting limit of 0.01 g/100 g. ^2^ d0, 14, 21-day 0 (colostrum), day 14 and day 21 of lactation milk samples. ^3^ SEM—standard error of the mean. ^4^ Total saturated-saturated fatty acids. ^5^ Total MUFA-monounsaturated fatty acids. ^6^ Total PUFA-polyunsaturated fatty acids. ^7^ Total n-3 PUFA-omega 3 polyunsaturated fatty acids. ^8^ Total n-6 PUFA-omega 6 polyunsaturated fatty acids. ^9^ n-6: n-3-ratio of omega 6 fatty acids to omega 3 fatty acids. ^a,b,c,d,e^ Means with different superscripts within a row are different (*p* < 0.05).

**Table 5:** Fatty acid composition (g/100g total fatty acids) of piglet blood plasma, adipose, liver and muscle tissue collected at weaning after offering sows diets containing soya or salmon oil and a low or phased dietary regimen during lactation.

Fatty Acid ^1^	Soya Oil		Salmon Oil		SEM ^4^	Oil	Energy	Oil × Energy
	Flat ^2^	Phased ^3^	Flat	Phased		*p*	*p*	*p*
Blood Plasma								
Total Saturated ^5^	42.6	41.4	41.3	45.3	1.375	0.348	0.295	0.073
Total MUFA ^6^	23.7	19.8	24.5	23.0	0.806	0.018	0.002	0.145
Total PUFA ^7^	33.5	38.4	33.7	31.3	1.838	0.069	0.539	0.055
Total n-3 PUFA ^8^	2.75	3.19	4.49	4.39	0.483	0.005	0.734	0.577
C18:3 (n-3)	0.97	1.2	1.23	1.3	0.104	0.094	0.167	0.445
C22:6 (n-3)	0.92	1.08	2.18	2.04	0.294	<0.001	0.988	0.620
Total n-6 PUFA ^9^	30.8 ^a^	35.2 ^b^	29.2 ^a^	26.9 ^a^	1.431	0.002	0.495	0.025
C18:2 (n-6)	25.4 ^a^	29.7 ^b^	24.8 ^a^	23.6 ^a^	1.01	0.002	0.138	0.011
C20:4 (n-6)	4.55	4.58	3.53	2.41	0.451	0.001	0.235	0.213
n6:n3 ^10^	12.8	12.7	7.4	7.2	1.01	<0.001	0.864	1.000
Adipose Tissue								
Total Saturated	35	34.2	36.2	35.5	0.593	0.044	0.226	0.93
Total MUFA	43	39.2	44.2	43.1	0.687	<0.001	0.001	0.065
Total PUFA	21.8 ^a^	26.3 ^b^	19.3 ^c^	21.2 ^a^	0.412	<0.001	<0.001	0.002
Total n-3 PUFA	2.15	2.42	2.47	3.11	0.131	<0.001	0.001	0.165
C18:3 (n-3)	1.63	2.04	1.55	1.87	0.051	0.02	<0.001	0.351
C20:5 (n-3)	0.06 ^a^	0.04 ^a^	0.12 ^b^	0.20 ^c^	0.017	<0.001	0.06	0.004
C22:5 (n-3)	0.19 ^a^	0.14 ^a^	0.31 ^b^	0.40 ^c^	0.032	<0.001	0.515	0.025
C22:6 (n-3)	0.12 ^a^	0.07 ^a^	0.32 ^b^	0.47 ^c^	0.047	<0.001	0.332	0.035
Total n-6 PUFA	19.6 ^a^	23.9 ^b^	16.8 ^c^	18.0 ^c^	0.448	<0.001	<0.001	0.001
C18:2 (n-6)	18.5 ^a^	22.8 ^b^	15.8 ^c^	17.0 ^c^	0.447	<0.001	<0.001	0.001
C20:4 (n-6)	0.31	0.32	0.28	0.27	0.013	0.003	0.878	0.413
n6:n3	9.44	9.88	6.84	6.09	0.434	<0.001	0.723	0.168
Liver Tissue								
Total Saturated	41.8 ^a^	39.8 ^b^	42.9 ^a,c^	43.6 ^c^	0.484	<0.001	0.205	0.009
Total MUFA	13.5	12.4	12.4	12.8	0.493	0.534	0.487	0.133
Total PUFA	44.4 ^a^	47.5 ^b^	44.4 ^a^	43.3 ^c^	0.368	<0.001	0.011	<0.001
Total n-3 PUFA	8.81	8.15	13.7	14.6	0.693	<0.001	0.874	0.263
C18:3 (n-3)	0.68	0.99	0.54	0.78	0.066	0.01	<0.001	0.578
C22:5 (n-3)	2.39 ^a,b^	2.15 ^a^	2.55 ^b^	2.84 ^c^	0.094	<0.001	0.737	0.008
C22:6 (n-3)	5.61	4.9	10.6	10.9	0.662	<0.001	0.758	0.449
Total n-6 PUFA	35.5 ^a^	39.3 ^b^	30.6 ^c^	28.6 ^c^	0.878	<0.001	0.308	<0.001
C18:2 (n-6)	19.5 ^a^	23.1 ^b^	16.5 ^c^	16.4 ^c^	0.507	<0.001	0.002	<0.001
C20:4 (n-6)	14.7	14.6	12.6	10.7	0.606	<0.001	0.111	0.146
n6:n3	4.37	4.88	2.25	2.17	0.274	<0.001	0.424	0.277
Muscle Tissue								
Total Saturated	36.7	36.1	38.2	37.6	0.589	0.014	0.331	0.954
Total MUFA	37.1	33.4	38.4	36.7	0.865	0.01	0.005	0.263
Total PUFA	26	30.5	23.3	25.5	0.592	<0.001	<0.001	0.06
Total n-3 PUFA	2.65	2.85	3.19	4.12	0.224	<0.001	0.016	0.113
C18:3 (n-3)	1.52	1.9	1.48	1.71	0.044	0.01	<0.001	0.096
C22:5 (n-3)	0.59	0.52	0.76	0.99	0.075	<0.001	0.269	0.061
C22:6 (n-3)	0.4 ^a^	0.30 ^a^	0.80 ^b^	1.25 ^c^	0.129	<0.001	0.192	0.036
Total n-6 PUFA	23.3 ^a^	27.7 ^b^	20.0 ^c^	21.3 ^c^	0.582	<0.001	<0.001	0.012
C18:2 (n-6)	20.4 ^a^	24.7 ^b^	17.5 ^c^	18.6 ^c^	0.463	<0.001	<0.001	0.001
C20:4 (n-6)	1.88	1.96	1.5	1.62	0.148	0.018	0.519	0.911
n6:n3	9.34	9.73	6.25	5.46	0.463	<0.001	0.663	0.212

^1^ Blood plasma fatty acids are reported as g/100 g of total fatty acids with a reporting limit of 1 g/100g. Adipose, liver and muscle tissue fatty acids are reported as g/100 g of total fatty acids with a reporting limit of 0.01 g/100 g. ^2^ 14.5 MJ/kg DE diet offered for 28 d of lactation. ^3^ 14.5 MJ/kg DE diet offered from day 1 to 14 of lactation and 15.5 MJ/kg DE diet offered from day 15 to 28 of lactation. ^4^ SEM—standard error of the mean. ^5^ Total saturated-saturated fatty acids. ^6^ Total MUFA-monounsaturated fatty acids. ^7^ Total PUFA-polyunsaturated fatty acids. ^8^ Total n-3 PUFA-omega 3 fatty acids polyunsaturated fatty acids. ^9^ Total n-6 PUFA-omega 6 fatty acids polyunsaturated fatty acids. ^10^ n-6: n-3-ratio of omega 6 fatty acids to omega 3 fatty acids. ^a,b,c^ Means with different superscripts within a row are different (*p* < 0.05).
